# Multi-parallel qPCR provides increased sensitivity and diagnostic breadth for gastrointestinal parasites of humans: field-based inferences on the impact of mass deworming

**DOI:** 10.1186/s13071-016-1314-y

**Published:** 2016-01-27

**Authors:** Alice V. Easton, Rita G. Oliveira, Elise M. O’Connell, Stella Kepha, Charles S. Mwandawiro, Sammy M. Njenga, Jimmy H. Kihara, Cassian Mwatele, Maurice R. Odiere, Simon J. Brooker, Joanne P. Webster, Roy M. Anderson, Thomas B. Nutman

**Affiliations:** Laboratory of Parasitic Diseases, NIAID, National Institutes of Health, Bethesda, MD USA; Department of Infectious Disease Epidemiology, Faculty of Medicine, Imperial College London, London, UK; School of Public Health, Makerere University College of Health Sciences, Kampala, Uganda; Eastern and Southern Africa Centre of International Parasite Control, Kenya Medical Research Institute, Nairobi, Kenya; Neglected Tropical Diseases Research Unit, Center for Global Health Research, Kenya Medical Research Institute, Kisumu, Kenya; Faculty of Infectious and Tropical Diseases, London School of Hygiene & Tropical Medicine, London, UK; Royal Veterinary College, University of London, Hertfordshire, UK

**Keywords:** qPCR, Diagnostics, Soil-transmitted helminths, Deworming, Impact evaluation, Gastrointestinal parasites, Co-infection, *Ascaris lumbricoides*

## Abstract

**Background:**

Although chronic morbidity in humans from soil transmitted helminth (STH) infections can be reduced by anthelmintic treatment, inconsistent diagnostic tools make it difficult to reliably measure the impact of deworming programs and often miss light helminth infections.

**Methods:**

Cryopreserved stool samples from 796 people (aged 2–81 years) in four villages in Bungoma County, western Kenya, were assessed using multi-parallel qPCR for 8 parasites and compared to point-of-contact assessments of the same stools by the 2-stool 2-slide Kato-Katz (KK) method. All subjects were treated with albendazole and all *Ascaris lumbricoides* expelled post-treatment were collected. Three months later, samples from 633 of these people were re-assessed by both qPCR and KK, re-treated with albendazole and the expelled worms collected.

**Results:**

Baseline prevalence by qPCR (*n* = 796) was 17 % for *A. lumbricoides*, 18 % for *Necator americanus*, 41 % for *Giardia lamblia* and 15 % for *Entamoeba histolytica*. The prevalence was <1 % for *Trichuris trichiura, Ancylostoma duodenale, Strongyloides stercoralis* and *Cryptosporidium parvum*. The sensitivity of qPCR was 98 % for *A. lumbricoides* and *N. americanus*, whereas KK sensitivity was 70 % and 32 %, respectively. Furthermore, qPCR detected infections with *T. trichiura* and *S. stercoralis* that were missed by KK, and infections with *G. lamblia* and *E. histolytica* that cannot be detected by KK. Infection intensities measured by qPCR and by KK were correlated for *A. lumbricoides* (r = 0.83, *p* < 0.0001) and *N. americanus* (r = 0.55, *p* < 0.0001). The number of *A. lumbricoides* worms expelled was correlated (*p* < 0.0001) with both the KK (r = 0.63) and qPCR intensity measurements (r = 0.60).

**Conclusions:**

KK may be an inadequate tool for stool-based surveillance in areas where hookworm or *Strongyloides* are common or where intensity of helminth infection is low after repeated rounds of chemotherapy. Because deworming programs need to distinguish between populations where parasitic infection is controlled and those where further treatment is required, multi-parallel qPCR (or similar high throughput molecular diagnostics) may provide new and important diagnostic information.

## Background

Efforts to control STH infections through mass drug administration (MDA), primarily through school-based deworming [1, 2], have increased in the past ten years. An estimated 325 million children were given preventative chemotherapy for STH in 2013 [3]. The goal of MDA for STH is to decrease transmission and reduce morbidity; in some areas elimination has also been proposed as a goal [1, 4]. The greatest reductions in parasite burdens occur at the beginning of the MDA programs, but reinfection occurs rapidly, and deworming must be repeated at regular intervals to sustain its impact [5–7]. As STH species are often co-endemic and treatable using the same drugs, programs to combat a range of helminths are increasingly being combined to create economies of scale [8–10].

The absence of reliable, inexpensive, and sensitive diagnostics to track changes in the prevalence and intensity of helminth infections after rounds of treatment has complicated efforts to assess the benefits of interventions [11, 12]. The KK method is commonly used in resource-limited settings because it is simple, quantitative, and can detect *Schistosoma spp*. and STH [13]. KK intensities serve as a proxy for worm burden, as previous studies based on worm expulsion for both *A. lumbricoides* and hookworm show strong correlations between egg output (KK) and worm load, modified by density dependence in parasite fecundity [14–16]. However, fecal egg count is an indirect and imperfect proxy for the intensity of infection [17].

KK requires a significant time investment by skilled parasitologists—and expertise in microscopy is increasingly rare. This method is highly susceptible to error [18]. Results depend on stool volume and consistency, which can vary by region, season, diet, age and day [19]. The highly time-sensitive detection of hookworm eggs by KK is particularly difficult [20, 21]. Clumping of eggs in stool could theoretically add to observed variability, and homogenization of fecal samples is recommended for detection of *Schistosoma mansoni* eggs, though evidence of clumping has not been found for *A. lumbricoides*, *T. trichiura* or hookworm eggs [22, 23].

The KK method is particularly unreliable in low prevalence settings such as the present study, as sensitivity for light infections is low [24–26]. A recent meta-analysis found that duplicate KK had 74-95 % sensitivity in high prevalence areas and 53–80 % in low prevalence areas for three STH species (*A. lumbricoides, T. trichiura* and hookworm) [27]. Alternatives to the standard KK diagnostic method exist, including direct smears, the McMaster, concentration methods and FLOTAC [28–30]. Among microscopy techniques for STH, FLOTAC is most sensitive for most species, and the agar plate method is most sensitive for *S. stercoralis* [30]. However, the version of FLOTAC designed for field-based use, the mini-FLOTAC, is comparable to KK [27, 31].

Quantitative polymerase chain reaction (qPCR)-based assays represent a promising alternative approach for the diagnosis of infection, epidemiological research on MDA impact and field-based central laboratory settings [32–34]. These have been developed for a large number of intestinal parasitic pathogens including STH (*A. lumbricoides, N. americanus, A. duodenale, T. trichiura, S. stercoralis),* protozoa (*G. lamblia, C. parvum/hominis, E. histolytica),* and *Schistosoma* species including *S. mansoni.* This makes it possible to distinguish between hookworm species, and between the pathogenic *E. histolytica* and the commensal *E. dispar* (identified as different species by [35]). Since *A. duodenale* has been found to have a greater impact on iron deficiency than *N. americanus* [36, 37], it is helpful for researchers and program implementers to know which species is prevalent in their target regions. For those trying to estimate burden based on egg count, it is also important to distinguish between species as output for the two hookworm species differs [38].

Although they are costly, these techniques are already used in centralized laboratories in many low- and middle-income countries. Molecular diagnostics for a wide range of infectious disease agents can improve sensitivity and specificity of measurements, allow for more accurate quantitation, increase throughput, require fewer trained personnel and offer insight into the relationships among multiple pathogens. From a practical perspective, qPCR assays may be particularly useful to monitor the intensity of STH (and other parasites) in areas nearing transmission elimination after repeated rounds of chemotherapy. They provide a more precise way to examine trends in low-intensity infection settings, determine if reinfection occurs post-treatment, and assess interruption of transmission in a defined setting.

This study was designed to measure the benefit of a novel molecular diagnostic tool relative to the traditional KK egg-counting technique commonly used in deworming impact evaluations. Our primary aim was to assess whether, and to what degree, high-throughput, multi-parallel qPCR increased sensitivity and diagnostic breadth compared to traditional stool-based parasitic diagnosis. A second aim was to assess whether qPCR could be used to measure the intensity of infection. As *A. lumbricoides* and *N. americanus* were the only STH present in the study setting beyond trace levels, this study focuses on these species.

## Methods

### Ethics statement

This study was approved by the Ethics Review Committee of the Kenya Medical Research Institute (Scientific Steering Committee protocol number 2688) and the Imperial College Research Ethics Committee (ICREC_ 13_1_15). Informed written consent was obtained from all adults and parents or guardians of each child. Minor assent was obtained from all children aged 12–17. Anyone found to be infected with any STH was treated with 400 mg albendazole during each phase of the study, and all previously-untreated village residents were offered albendazole at the end of each study phase.

### Participant recruitment and stool collection

Four villages were selected in the West Sang’alo administrative sub-location of Bungoma County in western Kenya. The population consists largely of a single subtribe of the Luhya community, and the primary occupation of most households is subsistence agriculture. This location was chosen based on high STH infection prevalence in past surveys [39]. Participants were recruited during house-to-house surveys, targeting all residents of the four study villages. Community health workers served as guides and translators. Geographical coordinates were recorded for each household. In total, 4778 people from 855 households were enrolled. A random number generator was used to select households for inclusion in the study. Stools were not collected from individuals residing in the households not selected, but these individuals were provided with albendazole at the end of each study phase.

Stool collection took place both at the schools serving the majority of study village residents and from designated community collection points. On days when collection was carried out at community collection points, targeted individuals were given containers and instructions the day prior to collection. Participants were asked to bring their samples to the collection point soon after they were made. On days where collection was carried out at primary schools, the collection pots were distributed directly to enrolled children based on enrollment records and the school roster, and children were asked to produce stools in the school latrines and return partially-filled containers to the study team. We aimed to collect stool from each participant on at least two days. Stool samples were analyzed on the day of collection using duplicate KK thick smears, 41.7 mg of stool each [40]. Duplicate slides from each sample were read by two different experienced readers. Technicians aimed to have stools processed within an hour of their preparation, and most slides were read within 1.5 h of preparation. Stools were not examined for protozoa in the field.

A 200 mg sample from each stool was stored at −15 without fixatives in a Precellys Soil grinding SK38 2 mL tube (Bertin Technologies, Montigny-le-Bretonneux, France) in the Bungoma County Hospital (Level 4) within 8 h of collection. Stools were shipped on dry ice to the National Institutes of Health (NIH) in Maryland, USA, and stored at −80 until processed.

### Treatment and worm expulsion

Following screening, all participants with a positive KK result for *A. lumbricoides*, along with 20 % additional subjects without *A. lumbricoides* (controls), were treated with 400 mg albendazole under direct observation. Stools from these participants were collected each morning on the second through the sixth day after treatment. This duration was chosen based on the results of a pilot test, which indicated that approximately 80 % of all worms expelled from an individual would be expelled during this timeframe [41–43]. In the field laboratory, *A. lumbricoides* worms were isolated daily from these stool samples by physically separating and collecting worms from fecal material. Worms were then washed, stored in tubes and cryopreserved.

In addition to all individuals who participated in the worm expulsion studies, all individuals who were positive for any STH infection, plus an additional 10 % of all other individuals (controls), were also treated with 400 mg albendazole. In total, 493 people were tested at this time-point. Everyone who was treated was asked to provide a stool sample three weeks post-treatment to examine treatment efficacy. After baseline data collection and direct treatment of these subgroups, treatment was provided to all children in the schools where surveys had been done and to all individuals in the four study villages.

Three months post-treatment, a follow-up parasitological survey and worm expulsion study were carried out using the same procedure as described above. Some people were present only at baseline or only at follow-up. There were 796 people with complete KK and qPCR records at baseline; 633 of these people also had complete records at follow-up. The total number of stools that were tested both by KK and by qPCR is 1884. This sample includes people who were present at only one time-point, stool samples from the efficacy time-point, and multiple stools from some individuals.

### Stool DNA extraction and qPCR

All reagents for DNA extraction were taken from the PowerSoil and PowerMag Kits (both produced by Mo Bio, Carlsbad, CA). The DNA extraction was an automated process based on PowerSoil reagents, using PowerMag magnetic beads and a magnetic plate (Alpaqua, Beverly, MA) to adapt the PowerSoil protocol for use on a Beckman Coulter Biomek NXP robot (Beckman Coulter, Brea, CA). Samples were homogenized on a Precellys 24 (Bertin Technologies, France) and processed in Costar 96-deep well assay blocks (Fisher Scientific, Waltham, MA). Each sample was spiked with 0.42 ng of an internal control plasmid to test for efficient extraction [44].

Following DNA extraction, 2 μL of the DNA sample was plated in quadruplicate wells of 384-well plates for qPCR. A master mix containing 2x TaqMan Fast Advanced Master Mix buffer and primers (final concentration 0.9 μM) and Fam-labeled probes (final concentration 0.25 μM) were added. DNA concentrations were calculated using a standard curve. The *A. lumbricoides*, *N. americanus* and *T. trichiura* positive controls were prepared using genomic DNA extracted from adult worms of these species. All other positive controls (for other helminths and protozoa) were plasmids constructed to match the target sequences of their associated primers and probes. The primers and probe sequences for all species except *N. americanus* and *T. trichiura* can be found in [32]. The *N. americanus* primer and probe sequences used were based on repeat sequences recently identified [Pilotte N, Papaiakovou M, Grant JR, Bierwert LA, Llewellyn S, McCarthy JS et al.: Improved PCR-Based Detection of Soil Transmitted Helminth Infections Using a Next-Generation Sequencing Approach to Assay Design, submitted]. The *T. trichiura* forward primer sequence is 5′-TTGCCTGTTGGGTGTATCTGTAA-3′, the reverse primer sequence is 5′-TGCTCATCCATCCGTTGGT-3′ and the probe sequence is 5′-FAM-TAAACTTCAAAATGCCC-3′ (GenBank accession no. HG805809.1). The plates were run on a ViiA7 (Life Technologies, Grand Island, NY) using parameters previously described [32].

### Controlled experiments to determine limit of detection for KK (Part A) and qPCR (Part B)

- Part A: Fifty slides were selected from the pool of slides prepared as part of the primary study. These 50 slides were read independently by five technicians in the field laboratory in Kenya. Thirty-four of these slides had *A. lumbricoides* eggs; the true number of eggs on each was estimated as the mean of all positive egg-counts by these 5 readers. In all other parts of this study, each slide was read only once (though each stool sample was made into two slides). The probability of detection was calculated as the proportion of all technicians who reported any eggs on each of these 34 slides. The number of eggs counted per slide by KK is multiplied by 24 to convert egg counts to EPG (since KK examines 41.7 mg of stool, and 1000/41.7 = 24).
- Part B: Egg-spiking experiments to measure qPCR sensitivity were performed by using eggs obtained from adult female worms. Briefly, an adult female worm from the worm expulsion phase of the study was dissected, and 2 cm of uterine tissue attached to the vaginal pore was added to 750 μL of lysis buffer (1 % SDS, 400 μg Proteinase K in H_2_O) to loosen the eggs from their sheath. The sheath was removed, the eggs were centrifuged and the supernatant was removed. The eggs were washed in distilled water and then re-suspended in water. Serial dilutions of the suspended eggs were made. From each dilution, 5 μL was pipetted onto a slide, and the eggs on that slide were read by microscopy. Three slides were made for each dilution to estimate the number of eggs in 5 μL at each dilution. Then, 5 μL of each dilution was pipetted into each of three Precellys Soil grinding tubes. These tubes were processed as described for DNA extraction. Each DNA extraction was plated for qPCR in quadruplicate. The proportion of wells in which qPCR detected an *A. lumbricoides* signal was taken as the probability of detection for that egg concentration. Since qPCR examined DNA extracted from 200 mg of stool, detecting 1 egg in 200 mg would be equivalent to detecting eggs from a stool with EPG 1000/200 = 5. Thus the number of eggs estimated to be in each tube was multiplied by 5 to calculate EPG.

### Statistical analysis

Statistical analysis was performed using Prism version 6.0 (GraphPad, La Jolla, CA), R version 3.2.1 (R Foundation for Statistical Computing, Vienna, Austria, 2015) and Winbugs [45]. Unless stated otherwise, arithmetic means were used for measurements of central tendency. The proportion of the population infected as measured by qPCR and KK were compared using the non-parametric Wilcoxon matched-pairs signed rank test, since STH infections are not normally distributed within the human population (the distributions are well described by the negative binomial probability model showing high degrees of parasite aggregation where the variance is greater than the mean) [46, 47]. Correlations were estimated from Spearman’s rank correlation coefficients in R. For comparisons of population proportions, only the people who had complete results from baseline and follow-up were included (*n* = 633). For comparisons of qPCR and KK results from the same stools, all stools with matched 2-slide KK and qPCR results are included, except those for which the internal control failed (*n* = 1869).

Sensitivity was calculated by classifying stool samples as positive by qPCR when cycle threshold values were below 40, and as positive by KK if an egg was seen on any of the two slides read per stool; a sample was considered “infected” if positive by either method. The high specificity of qPCR is reviewed in [48, 49] and additional evidence can be found in other studies [32, 50]. Kato Katz is generally also found to have very high specificity [24, 51, 52].

The sensitivity of tests for the detection of *A. lumbricoides* and *N. americanus* was also estimated by Bayesian latent class modeling, which does not assume perfect sensitivity of qPCR and KK, and assumes no knowledge of a latent variable: the true infection status of participants. The outcomes of the qPCR and KK tests (1 or 0) were treated as indicators of this latent variable. Maximum likelihood estimates for all parameters were calculated using Winbugs code [53]. For each simulation, four chains were run for one million iterations. A model accounting for covariance was used; conditional independence of KK and qPCR results was not assumed, as both tests depend on the same biological process to identify helminth infection (the production of eggs by worms in the intestine), and are shown in this study to be correlated. The following assumptions were included in the model: 95 % confidence that the specificity of each method was greater than 95 %, and 95 % confidence that the sensitivity of qPCR was greater than 80 %.

## Results

### Baseline prevalence of gastrointestinal parasites

**Fig. 1:**
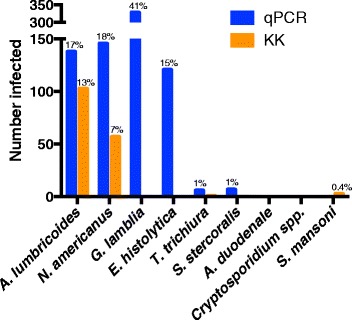
Baseline parasite prevalence by qPCR and KK. Number of people determined to be infected with each of the 8 parasites by qPCR (blue) or KK (orange) with the percentages listed above the bars

Since *A. lumbricoides* and hookworm infections were found in the study setting by microscopy, we sought to determine how using qPCR would change our understanding of *A. lumbricoides* and hookworm infection burden in the communities surveyed. Thus, the presence of 8 gastrointestinal parasites was determined by qPCR, and these prevalences were compared to those found by KK (in 796 individuals with complete records by both methods at study baseline). As shown in Fig. 1, at baseline the prevalence of *A. lumbricoides* was 17.3 % by qPCR (138/796), and 12.9 % by KK (103/796). The baseline prevalence of *N. americanus* was 18.3 % by qPCR (146/796), and 7.2 % by KK (57/796). All hookworm eggs observed by KK were assumed to be *N. americanus* as no *A. duodenale* DNA was identified by qPCR in any sample. Prevalence at baseline by the two methods is significantly different with *p* < 0.0001 for both *N. americanus* and *A. lumbricoides*. Based on the 633 samples with qPCR results from both baseline and follow-up, as seen in Table 1, the prevalence and intensity of both *A. lumbricoides* and *N. americanus* declined after treatment. This trend is apparent regardless of whether KK or qPCR is used. However, prevalence by qPCR is higher at both time points and mean intensity by qPCR never goes to zero.

**Table 1:** STH prevalence and intensity at baseline and at follow-up 3 months after albendazole therapy

	*A. lumbricoides*		*N. americanus*	
	Baseline	Follow-up	Baseline	Follow-up
Percent infected (by qPCR)	14	3	18	4
Percent infected (by KK)	9	1	6	1
Mean DNA concentration in stool (μg/μL)	15	1	301	14
Mean EPG	677	96	8	0

The percent of people infected with each species, and the mean intensity of infection, is measured at baseline and follow-up from the 633 individuals with KK and qPCR results at both time points. For qPCR, the mean intensity is measured in μg/μL, and for KK, it is measured in EPG

Based on 1869 samples tested by both methods, the sensitivity of qPCR was 98 % (261/266 positives) for *A. lumbricoides* and 98 % (234/238 positives) for *N. americanus.* For KK, these sensitivities were only 70 % (187/266) and 32 % (77/238). These figures are calculated based on all 1869 stool samples processed by both PCR and KK and assuming 100 % specificity by both methods. Samples were only considered positive if at least ¾ wells had a Ct below 40. Fewer than 1 % of positives for *A. lumbricoides* had a Ct >37. The average standard deviation of the 3–4 positive replicates for each positive sample was 0.46 Ct, meaning that quantitation replicates were largely consistent. Results were similar when calculated using latent class modeling: we calculate that qPCR for *A. lumbricoides* was 94 % sensitive (95 % CI 83–99 %) and 97 % specific (94–99 %), and KK testing for *A. lumbricoides* was 72 % sensitive (56–93 %) and 98 % specific (96–100 %). For *N. americanus*, we calculate that qPCR was 94 % sensitive (CI 83–99 %) and 97 % specific (93–99 %), and KK was 28 % sensitive (10–49 %) and 98 % specific (96–100 %).

**Fig. 2:**
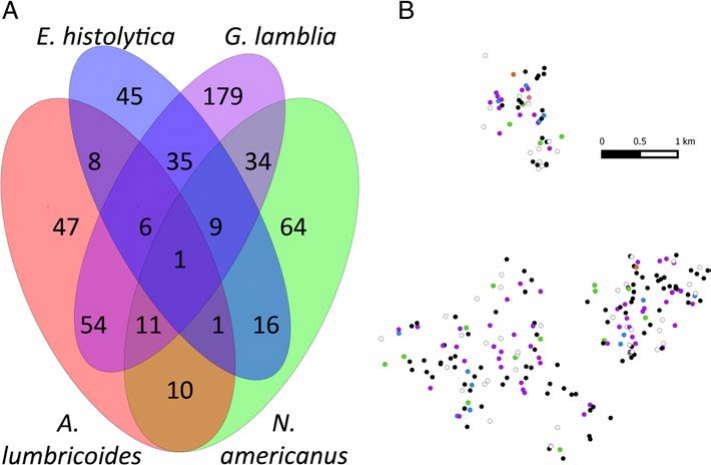
Polyparasitism is common among the study population. Panel **A** shows a Venn diagram related to infection with at least one of *A. lumbricoides, N. americanus, G. lamblia* and/or *E. histolytica* parasites. Panel **B** shows the household locations of those with single infections with *N. americanus* (green), S. *stercoralis* (pink), *T. trichiura* (orange), *G. lamblia* (purple) and *E. histolytica* (blue) or those households with more than one of these infections (black). Distance is depicted by the scaling ruler

The qPCR method also enabled us to test the same set of samples for other parasites, including gastrointestinal protozoa. Figure 1 shows that the study population has a high prevalence of *G. lamblia* (41 %, 329/796) and *E. histolytica* (15 %, 121/796). Polyparasitism was frequent (Fig. 2a) with varied parasite combinations. Interestingly, there was a positive relationship between the estimated intensities of *G. lamblia* and *A. lumbricoides* at baseline (r = 0.10, *p* = 0.007). When the parasitic infections were mapped to the households across the villages, there was neither obvious geographic clustering of any parasites (other than *A. lumbricoides,* shown separately in Fig. 6), nor clustering of the presence of polyparasitism as shown in Fig. 2b.

### Quantifying STH burden using qPCR

**Fig. 3:**
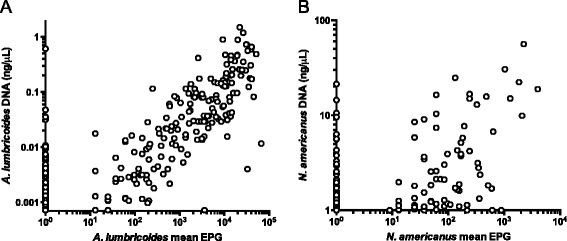
Relationship between qPCR and KK results for *A. lumbricoides* and *N. americanus*. Panel **A** shows the *A. lumbricoides* DNA concentration by qPCR as a function of the mean EPG measured by KK from the same stool across all time points (*n* = 1869). Panel **B** shows the *N. americanus* DNA concentration by qPCR as a function of the mean EPG measured by KK from the same stool

Since one of the benefits of KK is that it provides a quantitative assessment of the STH burden [13], we compared the quantitative output of KK and qPCR results from the same stool samples. Though many *A. lumbricoides* and *N. americanus* infections are missed by KK (Fig. 3) there was a strong positive correlation between the average egg count measured by KK and the average DNA concentration measured by qPCR (for *A. lumbricoides* r = 0.83, *p* < 0.0001; for *N. americanus* r = 0.55 *p* < 0.0001). The median *A. lumbricoides* DNA concentration for all positive qPCR results for *A. lumbricoides* was 0.013 ng/μL. We can see from Fig. 3a that the *A. lumbricoides* infections missed by KK are low-intensity by qPCR, below 0.013 ng/μL. However, the hookworm infections missed by KK cover a wide range of qPCR values, suggesting that they are a mixture of high and low intensity infections (Fig. 3b).

**Fig. 4:**
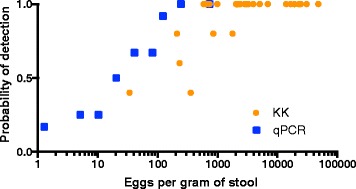
Parallel experiments calculate probability of *A. lumbricoides* detection by EPG. The probability that *A. lumbricoides* eggs will be detected by qPCR (in blue) or KK (in orange) is shown at different EPG values

Both qPCR and KK can more reliably detect heavy infections than light infections. Based on an egg-spiking experiment (see Methods), it can be seen from Fig. 4 that both qPCR and KK have a high probability of detecting an infected individual with a high EPG. However, at lower EPGs, the probability that a reader (KK) or a well (qPCR) will detect a signal decreases. At every EPG, the probability of detection is higher for qPCR than for KK until the EPG is so high that both have a very high probability of detecting an infection (Fig. 4).

### Response to albendazole treatment

**Fig. 5:**
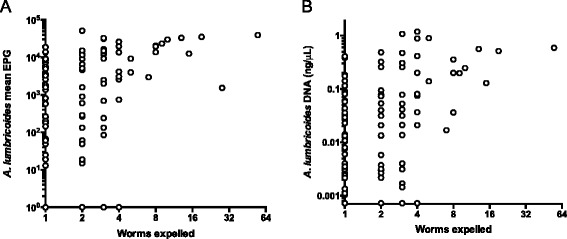
Relationship between KK and qPCR measurements and the number of *A. lumbricoides* worms expelled. For the set of 159 individuals who were part of the worm expulsion at baseline, their baseline average 2-stool duplicate KK result is plotted against the number of worms eventually collected from that person (Panel **A**). Points on the x-axis represent people who expelled worms but were negative for *A. lumbricoides* by KK, and points on the y-axis represent people who were positive for *A. lumbricoides* by KK but in whose stool worms were not found. Panel **B** shows the relationship between the *A. lumbricoides* DNA concentration calculated by qPCR and the number of expelled worms for the same set of 159 individuals

Infection intensities by KK and qPCR were each strongly correlated with the number of *A. lumbricoides* worms expelled following treatment at baseline (r = 0.63 for KK and 0.60 for qPCR, *p* < 0.0001, Fig. 5). As the number of worms increases, the KK and qPCR results appear to converge and plateau. Individuals with four or more worms had high measurements of egg intensity by both KK and qPCR, but intensity measurements from individuals with fewer than four worms covered the entire observed range of KK and qPCR measurements (Fig. 5). Of 90 individuals with positive KK and qPCR results at baseline, only 54 (60 %) ever expelled detectable *A. lumbricoides* worms. Since *A. lumbricoides* is a dioecious species, the sex of the worms in these low burden individuals will determine if eggs are produced and if they are fertilized.

**Fig. 6:**
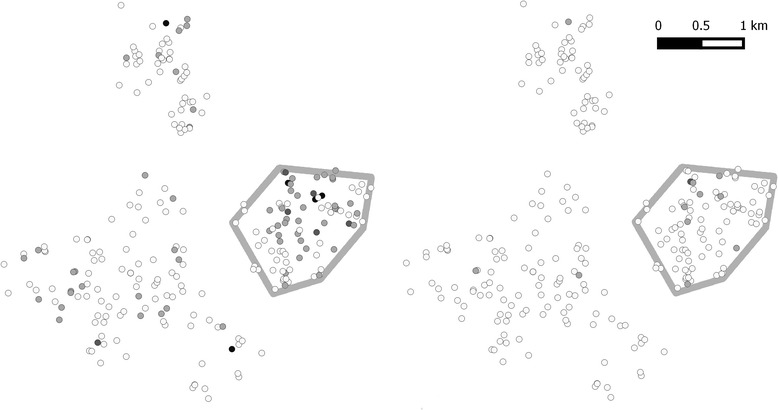
Intensity of *A. lumbricoides* infection by household at baseline and at follow-up three months after albendazole therapy. Households with *A. lumbricoides* DNA concentration (ng/μL) at baseline (left) and three months post-treatment (right). Households with no *A. lumbricoides* (white circles), light infections (<0.1 ng/μL, light gray circles), medium infections (0.1–0.2 ng/μL, dark gray circles) and heavy infections (>0.2 ng/μL, black circles) are shown. The village with the highest prevalence of individuals infected with *A. lumbricoides* at baseline and follow-up is outlined in gray

Three months after the baseline round of treatment, *A. lumbricoides* prevalence was reduced to 2.5 % (16/633) as measured by qPCR and 1.4 % (9/633) as measured by KK. Visualizing infections by household, there appears to be clustering in a single village (Fig. 6). While many infections were cleared by treatment, the area with the highest initial prevalence remained a focal area for reinfection after treatment. This village had a significantly higher prevalence at both baseline and follow-up when compared to the other three villages (z = 6.30, *p* < 0.00001 at baseline, and z = 3.04, *p* = 0.001 at follow-up). The prevalence of *N. americanus* was reduced at follow-up to 4.4 % (28/633) by qPCR and 1.1 % (7/633) by KK. As may be expected from a population with lower egg intensities, the sensitivity of KK was even lower at follow-up than it was at baseline: 53 % (9/17) for *A. lumbricoides* and 25 % (7/28) for *N. americanus*.

**Fig. 7:**
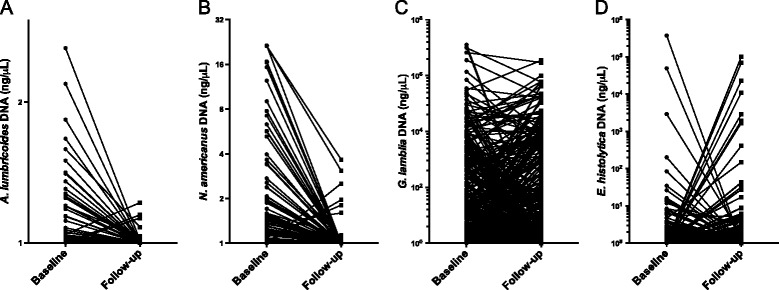
Infection intensities of individuals at baseline and at follow-up three months after albendazole therapy. Mean DNA concentrations, calculated from qPCR results, are shown for *A. lumbricoides* (Panel **A**), *N. americanus* (Panel **B**), *G. lamblia* (Panel **C**) and *E. histolytica* (Panel **D**) at baseline for 633 participants for whom paired samples were available. Each line represents a single individual before and three months after albendazole therapy

While albendazole treatment reduced the prevalence and intensity of *A. lumbricoides* and *N. americanus* infections (as seen by the reduction in dark circles in Fig. 6 and predominance of negative slopes in Fig. 7a and b), *G. lamblia* and *E. histolytica* prevalence by qPCR were substantial at both baseline and follow-up. As qPCR expanded our ability to measure other gastrointestinal parasite intensities from the same stool samples, we were able to examine whether the intensities of these protozoan infections changed following albendazole therapy, though changes were not expected. Though there was a transient drop in *G. lamblia* prevalence and intensity three weeks after treatment with albendazole (data not shown), there was no significant difference in *G. lamblia* and *E. histolytica* infection prevalence or intensity at follow-up (Fig. 7).

## Discussion

Diagnostic tools are crucial for mapping STH infections, designing programs that target hotspots of infection, and monitoring MDA program impact [17, 25, 54]. When STH infections are pushed toward local elimination in defined settings, it will become increasingly important to have highly sensitive diagnostic methods to assess whether transmission has ceased. Given the propensity for STH to bounce back to pre-control levels after chemotherapy cessation in the absence of other changes (such as significant improvements in hygiene and sanitation), sensitive diagnostic tools will be increasingly important as MDA programs expand to interrupt transmission. A parasitologist cannot identify a slide with an EPG lower than 24 (as EPG is calculated as 24 times the number of eggs seen on the slide). In contrast, qPCR using 200 mg of stool could theoretically identify an EPG as low as 5 (since one egg in 200 mg is 5 eggs in 1 g), or even less if DNA shed from eggs or worms was present in an egg-free 200 mg sample.

Despite working with a team of highly skilled parasitologists, we found that KK only detected 70 % of *A. lumbricoides* and 32 % of *N. americanus* infections detected by qPCR. False negatives from KK result from low rates of detection at low infection intensities, as seen in Fig. 4. It is possible that qPCR could identify DNA shed from worms in the intestines not producing eggs, though it is likely that most DNA measured by qPCR comes from eggs in stool [55]. A meta-analysis found that 1-sample duplicate KK resulted in 64.6 % sensitivity for *A. lumbricoides* and 63 % sensitivity for hookworm, and a 2-sample duplicate KK resulted in 69.2 % sensitivity for *A. lumbricoides* and 74.2 % for hookworm [27]. Our findings are in line with this meta-analysis for *A. lumbricoides* but lower for hookworm. Further, our data suggest that qPCR may be the “new gold standard,” to which KK should be compared.

Several previous studies have shown that PCR clearly outperforms microscopy for STH diagnosis [48]. In Malaysia, qPCR was found to be more sensitive than KK or a flotation technique [56]. Recently in Argentina, qPCR performed better than concentration microscopy or McMaster for all gastrointestinal parasites studied except *T. trichiura* [55]. A study in a low endemicity setting in Tanzania, in contrast, found that PCR results for hookworm were comparable with KK [57]. This might have been because their KK technique was exceptionally good (slides were examined exactly 20 min after preparation, which is difficult to achieve in a study where high throughput is required), or because the qPCR technique we used for hookworm is an improvement on what was previously available. Some of these differences could be related to different target sequences for qPCR. Our study used sequences from a variety of sources, based on head-to-head comparisons to identify those with the greatest sensitivity and specificity. Our study also has the strength of having an internal amplification control that allowed us to determine the robustness of our DNA extraction process. The absence of such a positive control was identified as a drawback in previous studies [32, 57]. It should be noted, however, that only 0.8 % of samples (15/1884) had to be removed from analysis due to failure of this internal control, indicating the reliability of our DNA extraction procedure.

A recent meta-analysis on the sensitivity of STH diagnostics concluded that there was not enough evidence on the sensitivity of PCR for STH to include it in the list of techniques evaluated [27]. The present study clearly shows, based on 1869 stool samples collected in the field and tested by KK and qPCR, that the latter method is considerably more sensitive for *A. lumbricoides* and *N. americanus* than two KK slides from a single stool (Fig. 3), and that significantly higher prevalences are calculated if qPCR is used rather than two KK slides from two stools (Fig. 1). The substantial difference between hookworm prevalence by qPCR and KK is comparable to that found using a similar qPCR method in Argentina, where prevalence was 37 % by qPCR compared to 21 % by microscopy [55].

One of the strengths of KK is that it provides a quantitative measure of infection burden. We have shown, based on 1869 stool samples (Fig. 3), that KK and qPCR measures are very strongly correlated for *A. lumbricoides* (r = 0.83, *p* <0.0001) and moderately correlated for *N. americanus* (r = 0.55, *p* <0.0001). By testing this relationship in a large field-based cohort, we were able to confirm findings from previous studies with fewer comparable measurements that *A. lumbricoides* and *N. americanus* qPCR and KK results are correlated [32, 55, 57]. The fact that the quantitative measures from the KK and qPCR tests are highly correlated indicates that DNA concentrations calculated against a standard curve for qPCR can be used as a quantitative measure of infection intensity.

EPG is used as a proxy for worm burden and hence as an assessment of transmission potential in defined populations [58, 59]. We have shown that EPG and DNA concentration are equally good predictors of worm burden, with similar correlation coefficients (Fig. 5). We have shown evidence of density-dependent egg production, since EPG and DNA concentration plateau at high levels of worm burden as found in [14, 47, 60]. However, the fact that people with fewer than five worms can have a qPCR or KK measurement covering the entire observed range suggests that the approach of estimating worm burden from EPG or qPCR should be used with caution.

There is increased pressure to combine disease control programs to save costs, given that many people suffer from gastrointestinal polyparasitism in resource-limited settings [61, 62]. The particular nature of a community’s multiple overlapping infections is important when selecting drugs for intervention [63]. As found in previous studies, qPCR allowed us to expand the range of parasites identified in the study setting and to identify the distribution of polyparasitism. As clearance of helminths may perturb the balance of infective agents, possibly allowing for increased colonization by some protozoan species [64], it may be important to track changes in colonization by other pathogens following MDA.

One possible drawback to this study is that we did not collect clinical information on infection with *E. histolytica*, *G. lamblia* or *S. stercoralis*. Neither did we examine stools using highly sensitive methods for these parasites (agar plate for *S. stercoralis*, coproantigen detection for *G. lamblia* and *E. histolytica*).

## Conclusions

Based on the present study, the prevalence of STH is likely to be underestimated by current mapping efforts in many low endemicity settings. As an alternative, qPCR can be used to more accurately assess changes in infection prevalence and intensity following MDA. This is particularly important as periodic MDA continues to bring down the prevalence of STH. Although qPCR requires transporting samples to reference laboratories where they can be processed, the use of such molecular techniques allows for more sensitive measurements of infection prevalence and intensity. Multi-parallel qPCR for STH can be more easily undertaken in resource-limited settings than can multiplex qPCR, as more standard (and accessible) instrumentation can be used [32]. KK still serves an important purpose for mapping areas of presumed high STH and *Schistosoma spp.* prevalence, but thorough examinations of the comparative costs of qPCR and KK under field conditions are necessary. Though monitoring and evaluation teams are currently asking whether they can afford to use qPCR for STH surveillance, they may soon be asking whether they can afford to lose so much information by putting off building their capacity in modern molecular methods.

## Abbreviations

STH: Soil transmitted helminth
MDA: Mass drug administration
KK: Kato-Katz
qPCR: Quantitative (or real-time) polymerase chain reaction

## References

[1] Webster JP, Molyneux DH, Hotez PJ, Fenwick A (2014). The contribution of mass drug administration to global health: past, present and future. Philos Trans R Soc Lond B Biol Sci.

[2] Pullan RL, Brooker SJ (2012). The global limits and population at risk of soil-transmitted helminth infections in 2010. Parasit Vectors.

[3] WHO (2015). Soil-transmitted helminthiases: number of children treated in 2013: World Health Organization.

[4] Croce D, Porazzi E, Foglia E, Restelli U, Sinuon M, Socheat D (2010). Cost-effectiveness of a successful schistosomiasis control programme in Cambodia (1995–2006). Acta Trop.

[5] Lustigman S, Prichard RK, Gazzinelli A, Grant WN, Boatin BA, McCarthy JS (2012). A research agenda for helminth diseases of humans: the problem of helminthiases. PLoS Negl Trop Dis.

[6] Anderson R, Truscott J, Hollingsworth TD (2014). The coverage and frequency of mass drug administration required to eliminate persistent transmission of soil-transmitted helminths. Philos Trans R Soc Lond B Biol Sci.

[7] Jia TW, Melville S, Utzinger J, King CH, Zhou XN (2012). Soil-transmitted helminth reinfection after drug treatment: a systematic review and meta-analysis. PLoS Negl Trop Dis.

[8] Brooker S, Kabatereine NB, Fleming F, Devlin N (2008). Cost and cost-effectiveness of nationwide school-based helminth control in Uganda: intra-country variation and effects of scaling-up. Health Policy Plan.

[9] Clements AC, Deville MA, Ndayishimiye O, Brooker S, Fenwick A (2010). Spatial co-distribution of neglected tropical diseases in the east African great lakes region: revisiting the justification for integrated control. Trop Med Int Health.

[10] Prichard RK, Basanez MG, Boatin BA, McCarthy JS, Garcia HH, Yang GJ (2012). A research agenda for helminth diseases of humans: intervention for control and elimination. PLoS Negl Trop Dis.

[11] Brooker S, Kabatereine NB, Gyapong JO, Stothard JR, Utzinger J (2009). Rapid mapping of schistosomiasis and other neglected tropical diseases in the context of integrated control programmes in Africa. Parasitology.

[12] Boatin BA, Basanez MG, Prichard RK, Awadzi K, Barakat RM, Garcia HH (2012). A research agenda for helminth diseases of humans: towards control and elimination. PLoS Negl Trop Dis.

[13] Katz N, Chaves A, Pellegrino J (1972). A simple device for quantitative stool thick-smear technique in Schistosomiasis mansoni. Rev Inst Med Trop Sao Paulo.

[14] Elkins DB, Haswell-Elkins M, Anderson RM (1986). The epidemiology and control of intestinal helminths in the Pulicat Lake region of Southern India. I. Study design and pre- and post-treatment observations on Ascaris lumbricoides infection. Trans R Soc Trop Med Hyg.

[15] Bundy DA, Hall A, Medley GF, Savioli L (1992). Evaluating measures to control intestinal parasitic infections. World Health Stat Q.

[16] Anderson RM, Schad GA (1985). Hookworm burdens and faecal egg counts: an analysis of the biological basis of variation. Trans R Soc Trop Med Hyg.

[17] McCarthy JS, Lustigman S, Yang GJ, Barakat RM, Garcia HH, Sripa B (2012). A research agenda for helminth diseases of humans: diagnostics for control and elimination programmes. PLoS Negl Trop Dis.

[18] Levecke B, Behnke JM, Ajjampur SS, Albonico M, Ame SM, Charlier J (2011). A comparison of the sensitivity and fecal egg counts of the McMaster egg counting and Kato-Katz thick smear methods for soil-transmitted helminths. PLoS Negl Trop Dis.

[19] Stoll NR. Investigations on the control of hookworm disease. XXXIII. The significance of egg count data in *Necator americanus* infestations. American Journal of Hygiene. 1924;4:466-500.

[20] Engels D, Nahimana S, Gryseels B (1996). Comparison of the direct faecal smear and two thick smear techniques for the diagnosis of intestinal parasitic infections. Trans R Soc Trop Med Hyg.

[21] Santos FL, Cerqueira EJ, Soares NM (2005). Comparison of the thick smear and Kato-Katz techniques for diagnosis of intestinal helminth infections. Rev Soc Bras Med Trop.

[22] Ye XP, Donnelly CA, Fu YL, Wu ZX (1997). The non-randomness of the distribution of Trichuris trichiura and Ascaris lumbricoides eggs in faeces and the effect of stirring faecal specimens. Trop Med Int Health.

[23] Krauth SJ, Coulibaly JT, Knopp S, Traore M, N’Goran EK, Utzinger J (2012). An in-depth analysis of a piece of shit: distribution of Schistosoma mansoni and hookworm eggs in human stool. PLoS Negl Trop Dis.

[24] Knopp S, Mgeni AF, Khamis IS, Steinmann P, Stothard JR, Rollinson D (2008). Diagnosis of soil-transmitted helminths in the era of preventive chemotherapy: effect of multiple stool sampling and use of different diagnostic techniques. PLoS Negl Trop Dis.

[25] Bergquist R, Johansen MV, Utzinger J (2009). Diagnostic dilemmas in helminthology: what tools to use and when?. Trends Parasitol.

[26] Lamberton PH, Kabatereine NB, Oguttu DW, Fenwick A, Webster JP (2014). Sensitivity and specificity of multiple Kato-Katz thick smears and a circulating cathodic antigen test for Schistosoma mansoni diagnosis pre- and post-repeated-praziquantel treatment. PLoS Negl Trop Dis.

[27] Nikolay B, Brooker SJ, Pullan RL (2014). Sensitivity of diagnostic tests for human soil-transmitted helminth infections: a meta-analysis in the absence of a true gold standard. Int J Parasitol.

[28] Knopp S, Rinaldi L, Khamis IS, Stothard JR, Rollinson D, Maurelli MP (2009). A single FLOTAC is more sensitive than triplicate Kato-Katz for the diagnosis of low-intensity soil-transmitted helminth infections. Trans R Soc Trop Med Hyg.

[29] Jeandron A, Abdyldaieva G, Usubalieva J, Ensink JH, Cox J, Matthys B (2010). Accuracy of the Kato-Katz, adhesive tape and FLOTAC techniques for helminth diagnosis among children in Kyrgyzstan. Acta Trop.

[30] Glinz D, Silue KD, Knopp S, Lohourignon LK, Yao KP, Steinmann P (2010). Comparing diagnostic accuracy of Kato-Katz, Koga agar plate, ether-concentration, and FLOTAC for Schistosoma mansoni and soil-transmitted helminths. PLoS Negl Trop Dis.

[31] Assefa LM, Crellen T, Kepha S, Kihara JH, Njenga SM, Pullan RL (2014). Diagnostic accuracy and cost-effectiveness of alternative methods for detection of soil-transmitted helminths in a post-treatment setting in western Kenya. PLoS Negl Trop Dis.

[32] Mejia R, Vicuna Y, Broncano N, Sandoval C, Vaca M, Chico M (2013). A novel, multi-parallel, real-time polymerase chain reaction approach for eight gastrointestinal parasites provides improved diagnostic capabilities to resource-limited at-risk populations. Am J Trop Med Hyg.

[33] Taniuchi M, Verweij JJ, Noor Z, Sobuz SU, Lieshout L, Petri WA (2011). High throughput multiplex PCR and probe-based detection with Luminex beads for seven intestinal parasites. Am J Trop Med Hyg.

[34] ten Hove RJ, van Esbroeck M, Vervoort T, van den Ende J, van Lieshout L, Verweij JJ (2009). Molecular diagnostics of intestinal parasites in returning travellers. Eur J Clin Microbiol Infect Dis.

[35] Diamond LS, Clark CG (1993). A redescription of Entamoeba histolytica Schaudinn, 1903 (Emended Walker, 1911) separating it from Entamoeba dispar Brumpt, 1925. J Eukaryot Microbiol.

[36] Jonker FA, Calis JC, Phiri K, Brienen EA, Khoffi H, Brabin BJ (2012). Real-time PCR demonstrates Ancylostoma duodenale is a key factor in the etiology of severe anemia and iron deficiency in Malawian pre-school children. PLoS Negl Trop Dis.

[37] Albonico M, Stoltzfus RJ, Savioli L, Tielsch JM, Chwaya HM, Ercole E (1998). Epidemiological evidence for a differential effect of hookworm species, Ancylostoma duodenale or Necator americanus, on iron status of children. Int J Epidemiol.

[38] Hoagland KE, Schad GA (1978). Necator americanus and Ancylostoma duodenale: life history parameters and epidemiological implications of two sympatric hookworms of humans. Exp Parasitol.

[39] Pullan RL, Gething PW, Smith JL, Mwandawiro CS, Sturrock HJ, Gitonga CW (2011). Spatial modelling of soil-transmitted helminth infections in Kenya: a disease control planning tool. PLoS Negl Trop Dis.

[40] Katz N, Chaves A, Pellegrino J (1972). A simple device for quantitative stool thick smear technique in *Schistosomiasis mansoni*. Revista do Instituto Medicina Tropical de Sao Paulo.

[41] Williams-Blangero S, Subedi J, Upadhayay RP, Manral DB, Rai DR, Jha B (1999). Genetic analysis of susceptibility to infection with Ascaris lumbricoides. Am J Trop Med Hyg.

[42] Forrester JE, Scott ME (1990). Measurement of Ascaris lumbricoides infection intensity and the dynamics of expulsion following treatment with mebendazole. Parasitology.

[43] Bundy DA, Thompson DE, Cooper ES, Blanchard J (1985). Rate of expulsion of Trichuris trichiura with multiple and single dose regimens of albendazole. Trans R Soc Trop Med Hyg.

[44] Deer DM, Lampel KA, Gonzalez-Escalona N (2010). A versatile internal control for use as DNA in real-time PCR and as RNA in real-time reverse transcription PCR assays. Lett Appl Microbiol.

[45] Lunn DJ, Thomas A, Best N, Spiegelhalter D (2000). WinBUGS - A Bayesian modelling framework: Concepts, structure, and extensibility. Stat Comput.

[46] Guyatt HL, Bundy DA, Medley GF, Grenfell BT (1990). The relationship between the frequency distribution of Ascaris lumbricoides and the prevalence and intensity of infection in human communities. Parasitology.

[47] Anderson RM, May RM (1985). Helminth infections of humans: mathematical models, population dynamics, and control. Adv Parasitol.

[48] Verweij JJ (2014). Application of PCR-based methods for diagnosis of intestinal parasitic infections in the clinical laboratory. Parasitology.

[49] Stensvold CR, Lebbad M, Verweij JJ (2011). The impact of genetic diversity in protozoa on molecular diagnostics. Trends Parasitol.

[50] Verweij JJ, Blange RA, Templeton K, Schinkel J, Brienen EA, van Rooyen MA (2004). Simultaneous detection of Entamoeba histolytica, Giardia lamblia, and Cryptosporidium parvum in fecal samples by using multiplex real-time PCR. J Clin Microbiol.

[51] Booth M, Vounatsou P, N’Goran EK, Tanner M, Utzinger J (2003). The influence of sampling effort and the performance of the Kato-Katz technique in diagnosing Schistosoma mansoni and hookworm co-infections in rural Cote d’Ivoire. Parasitology.

[52] Ebrahim A, El-Morshedy H, Omer E, El-Daly S, Barakat R (1997). Evaluation of the Kato-Katz thick smear and formol ether sedimentation techniques for quantitative diagnosis of Schistosoma mansoni infection. Am J Trop Med Hyg.

[53] Branscum AJ, Gardner IA, Johnson WO (2005). Estimation of diagnostic-test sensitivity and specificity through Bayesian modeling. Prev Vet Med.

[54] Harhay MO, Horton J, Olliaro PL, Utzinger J (2011). Diagnostics are central for a truly holistic approach against intestinal parasitic diseases. Int J Infect Dis.

[55] Cimino RO, Jeun R, Juarez M, Cajal PS, Vargas P, Echazu A (2015). Identification of human intestinal parasites affecting an asymptomatic peri-urban Argentinian population using multi-parallel quantitative real-time polymerase chain reaction. Parasit Vectors.

[56] Basuni M, Muhi J, Othman N, Verweij JJ, Ahmad M, Miswan N (2011). A pentaplex real-time polymerase chain reaction assay for detection of four species of soil-transmitted helminths. Am J Trop Med Hyg.

[57] Knopp S, Salim N, Schindler T, Karagiannis Voules DA, Rothen J, Lweno O (2014). Diagnostic accuracy of Kato-Katz, FLOTAC, Baermann, and PCR methods for the detection of light-intensity hookworm and Strongyloides stercoralis infections in Tanzania. Am J Trop Med Hyg.

[58] Holland CV, Asaolu SO, Crompton DW, Stoddart RC, Macdonald R, Torimiro SE (1989). The epidemiology of Ascaris lumbricoides and other soil-transmitted helminths in primary school children from Ile-Ife, Nigeria. Parasitology.

[59] Croll NA, Anderson RM, Gyorkos TW, Ghadirian E (1982). The population biology and control of Ascaris lumbricoides in a rural community in Iran. TRSTMH.

[60] Martin J, Keymer A, Isherwood RJ, Wainwright SM (1983). The prevalence and intensity of Ascaris lumbricoides infections in Moslem children from northern Bangladesh. Trans R Soc Trop Med Hyg.

[61] Pearson RD (2002). An Update on the Geohelminths: Ascaris lumbricoides, Hookworms, Trichuris trichiura, and Strongyloides stercoralis. Curr Infect Dis Rep.

[62] Mupfasoni D, Karibushi B, Koukounari A, Ruberanziza E, Kaberuka T, Kramer MH (2009). Polyparasite helminth infections and their association to anaemia and undernutrition in Northern Rwanda. PLoS Negl Trop Dis.

[63] Hotez P (2011). Enlarging the “Audacious Goal”: elimination of the world’s high prevalence neglected tropical diseases. Vaccine.

[64] Knowles SC, Fenton A, Petchey OL, Jones TR, Barber R, Pedersen AB (2013). Stability of within-host-parasite communities in a wild mammal system. Proc Biol Sci.

